# Supplementary material for: Extraction of accurate cytoskeletal actin velocity distributions from noisy measurements
Source: Nat Commun. 2022 Aug 13;13:4749. doi: 10.1038/s41467-022-31583-y (PMC9376101; doi:10.1038/s41467-022-31583-y)
Supplement: Supplementary file 1 — Supplementary Information [file 41467_2022_31583_MOESM1_ESM.pdf]

Supplementary information for: Extraction of accurate cytoskeletal actin velocity distributions from noisy measurements

Cayla M. Miller[a], **Elgin Korkmazhan**[b], **and Alexander R. Dunn**[a,c]✉

[a]Department of Chemical Engineering, Stanford University, Stanford, CA, USA
[b]Biophysics Program, Stanford University, Stanford, CA, USA
[c]Stanford Cardiovascular Institute, Stanford University School of Medicine, Stanford, CA, USA

Supplementary Note 1: Derivation of distance distribution

The analytical form for the distribution of measured distances, d, given a true distance, s, is an extension of the derivation by Churchmann et al.(1). If we assume that two points are located at (0,0) and (s,0), and that the measured coordinates are Gaussian distributed around the true location with variance σ^2, we can say that $x_1, y_1, y_2 \sim N(0, \sigma^2)$ and $x_2 \sim N(s, \sigma^2)$. As a result, the measured distance along x and y, d_x and d_y, respectively, are also normally distributed.

$$d_x = x_2 - x_1 \sim N(s, 2\sigma^2)$$

$$d_y = y_2 - y_1 \sim N(0, 2\sigma^2)$$

The noncentral χ distribution describes the distribution of a random variable z given by

$$z = \sqrt{\sum_i^k \left(\frac{X_i}{\sigma_i}\right)^2}$$

where X_i are normally distributed random variables with means μ_i and variances σ_i^2.
Therefore we may define z in terms of d as a noncentral χ-distributed random variable with k=2:

$$z = \sqrt{\left(\frac{d_x}{\sigma_{dx}}\right)^2 + \left(\frac{d_y}{\sigma_{dy}}\right)^2} = \frac{d}{\sqrt{2}\sigma}$$

Assuming σ_{dx} and σ_{dy} are equal, the noncentral χ distribution is

$$f_z(z,k,\lambda) = \lambda(\lambda z)^{-k/2} exp\left(-\frac{z^2+\lambda^2}{2}\right)\left(z^k\right) I_{k/2-1}(\lambda z),$$

where $I_{k/2-1}$ is a modified Bessel function of the first kind and

$$\lambda = \sqrt{\sum_i^k \left(\frac{\mu_i}{\sigma_i}\right)^2} = \sqrt{\frac{s^2}{2\sigma^2} + \frac{0^2}{2\sigma^2}} = \frac{s}{\sqrt{2}\sigma}.$$

Dividing by $\sqrt{2}\sigma$ to determine the distribution of d, and substituting $k = 2$ and $\lambda = s/(\sqrt{2}\sigma)$, we find that

$$f_d(d,s,\sigma) = \frac{d}{2\sigma^2} exp\left(-\frac{1}{4\sigma^2}\left(d^2+s^2\right)\right) I_0\left(\frac{ds}{2\sigma^2}\right)$$

Supplementary Note 2: Numerical approximation of Bayes Theorem (Eqn. 1)

The probability of observing a step distance, d, given a distribution of true distances, $f_s(s)$, is given by

$$f_d(d) = \int_{-\infty}^{\infty} f_d(d|s=S) f_s(S) dS. \quad \textbf{(1)}$$

While this equation may be expressed analytically for some forms of $f_s(s)$, we fit to a numerical approximation of this equation for consistency and ease of application to various functional forms. For our case, where we fit to distances, no values are less than zero, and the limits become zero to infinity. We approximate this integral as a Riemann sum over the interval from 0 to 1000 nm, with 100 linearly-spaced bin edges (bin width = 10 nm). For each bin, both $f_s(S)$ and $f_d(d|s=S)$ are calculated at both bin edges, averaged, and multiplied by the bin width. These are summed to give $f_d(d)$.
For the jump process as described in Supplementary Note 3, Bayes rule is not defined exactly as above due to the discrete discontinuity at $s = 0$. Instead, because the probability is defined piece-wise (Eqn. 4), Bayes theorem is rewritten in this case to give

$$f_d(d) = f_d(d|s=0) f_s(s=0) + \int_{0+}^{\infty} f_d(d|s=S) f_s(S) dS. \quad \textbf{(2)}$$

We approximate the integral here by approaching zero from the right, using 100 linearly-spaced bin edges from 0.0001 to 1000 nm.

Supplementary Note 3: Drift-diffusion and jump process models

A. Drift-diffusion. We modeled F-actin movement with two simple drift-diffusion models that we will collectively call the drift-diffusion model. In the first model, the particles undergo 1-D Brownian motion while drifting at a constant 1-D velocity. This results in folded Gaussian distributed distances, with location parameter μ equal to the drift velocity times the observation time and spread parameter σ^2 equal to the diffusion coefficient times observation time. The second model differs from the first only in that the particles are allowed to diffuse in 2-D while drifting in 1-D, which results in Rice-distributed distances with center ν equal to the drift velocity times the observation time and spread parameter σ^2 equal to the diffusion coefficient times observation time.

B. Position jump. We also modeled F-actin movement with a simple one dimensional unidirectional jump process with independent identically distributed (IID) exponential wait times between jumps and IID exponential jump distances at jumps. This function is sufficiently simple that we can obtain the solution in real space. When observed at a time interval τ, the total number of jumps in the interval will be Poisson distributed with mean $\mu\tau$ where μ is the jump rate.
If k non-zero jumps occur in an interval, the total distance moved in that interval will be distributed as the sum of k IID exponential random variables each with some rate parameter β. This gives a Gamma distribution of distances with shape parameter k and rate parameter β.
To find the probability distribution of the distance s moved for a given observation time window τ, we first need to sum up the probability that there is one jump of size s; two jumps of total size s; three jumps of total size s; and so on, giving

$$\begin{aligned} &\sum_{k=1}^{\infty} \frac{(\mu\tau)^k}{k!} e^{-\mu\tau} \gamma(k,\beta) \\ &= \sum_{k=1}^{\infty} \frac{(\mu\tau)^k}{k!} e^{-\mu\tau} e^{-\beta s} s^{k-1} \frac{\beta^k}{(k-1)!} \\ &= \frac{1}{s} e^{-\mu\tau-\beta s} \sum_{k=1}^{\infty} \frac{(\mu\tau\beta s)^k}{k!(k-1)!} \\ &= \frac{1}{s} e^{-\mu\tau-\beta s} \sqrt{\mu\tau\beta s} I_1(2\sqrt{\mu\tau\beta s}) \\ &= e^{-\mu\tau-\beta s} \sqrt{\frac{\mu\tau\beta}{s}} I_1(2\sqrt{\mu\tau\beta s}) \end{aligned} \tag{3}$$

where $\gamma(k,\beta)$ is the Gamma distribution with shape parameter k, rate parameter β and I_1 is the modified Bessel function of the first kind, of order 1. This expression is incomplete since we also need to consider the (discrete) probability of moving a distance of exactly $s = 0$, corresponding to the case of no jumps. The probability of a point moving exactly zero distance is equal to the probability that a jump does not occur in the given observation interval, such that $P(s = 0) = e^{-\mu\tau}$. Therefore, the complete normalized probability distribution for moving a distance $s \geq 0$ is defined piece-wise as

$$P(s) = \begin{cases} e^{-\mu\tau} \text{ for } s = 0 \\ e^{-\mu\tau-\beta s} \sqrt{\frac{\mu\tau\beta}{s}} I_1(2\sqrt{\mu\tau\beta s}) \text{ for } s > 0 \end{cases} \tag{4}$$

C. Position jump with reversals. To model 1D position jumps with reversals, we keep the properties above (IID exponentially distributed jump distances and IID exponentially distributed wait times between jumps), but add a constant probability of reversing direction on any jump.
This model was not readily tractable in real space, and instead fitting to this model is done via iterative Monte Carlo simulation (see Methods).

Supplementary Note 4: Estimation of F-actin lifetimes

In order to estimate the lifetimes of our measured F-actin populations, we analyzed the lifetimes of our tracked F-actin puncta. We begin with the fixed-cell puncta as a baseline of non-turnover based lifetimes (i.e. lifetimes arising from SiR-actin binding kinetics, bleaching, etc.). Lifetimes in fixed cells were well fit by a biexponential with rates $r_1 = 0.06/s$ and $r_2 = 0.33/s$ (Supplementary Fig. 11, left). While typical reversible binding is expected to yield only a single exponential, these two rates may arise from SiR-actin (based on the actin binding moiety from jasplakinolide) binding to F-actin in at least two modes, potentially dependent on F-actin nucleotide state(2).

We then fit the puncta lifetimes measured in live cells to a biexponential. Because these rates are expected to arise from the combined rates of bleaching, SiR-actin unbinding and F-actin turnover, the two rates are given by $r_1 + r_{to}$ and $r_2 + r_{to}$, where r_1 and r_2 are the rates from the fixed population fits, and r_{to} is the turnover rate of speckles in a given population. These fits yielded F-actin turnover rates of 0.06/s for cortical F-actin and 0.03/s for stress fiber F-actin, giving lifetimes of 18 and 34 s, respectively (Supplementary Fig. 11, middle and right). These turnover rates represent turnover both from depolymerization and movement out of the TIRF excitation field, and are comparable with previous FRAP measurements(3–6).

Supplementary Note 5: Robustness testing

We analyzed and compared subsets of the data to probe the possible effects of various forms of error from particle tracking. First, to test whether high speckle density in certain regions of the cell might lead to tracking errors (e.g. false linkages from overlapping fluorophores), we compared measured displacements from high density images to measured displacements from low density images. Because the SiR-actin signal bleached significantly during the acquisition, displacements measured during varying time periods of the movies (Supplementary Fig. 12) represent measurements at varying speckle densities. We quantified these displacements in live cells both at the 2 s timescale (Supplementary Fig. 12a) and the 10 s timescale (Supplementary Fig. 12b), where displacements became more easily distinguishable from localization error. In both cases, the distributions were in good agreement with one another throughout the movies. These data suggest that the particle tracking approach used here is robust to variations in fluorophore density.

As an independent test of whether high speckle density regions have greater tracking errors, we compared measured displacements of the brightest 25%, the dimmest 25% and the remaining 50% of tracks, where brightness was determined from the average amplitude of the best fit Gaussian during subpixel localization. These displacements were greatest for the dimmest tracks and least for the brightest tracks, likely because dim fluorophores were more poorly localized than bright ones. We tested the robustness of our fits by fitting each of these brightness populations to a Weibull distribution, though without updating our noise model to more accurately reflect the localization error of each subset of tracks. Best fits to these data yielded shape parameters between 1 and 1.5, consistent with motion being more Poisson-like than diffusive, and similar shape parameters for all three brightness populations (Supplementary Fig. 13).

We further visualized the variation in position (another measure of localization error) of tracks in fixed cells, to determine whether there was a spatial pattern in localization error (Supplementary Fig. 14). Instead, we found that the variation of localization errors is present throughout the cell, without any clear segregation of high or low localization errors to certain regions of the cell.

Last, we compared fits from tracks of varying lifetimes. For any given time between a pair of localizations (e.g. 8 s apart), measured displacements may come from tracks of varying lengths: those that last only the given time delay, and those that last much longer. When fit to a Weibull, we found that for a given observation timescale, tracks of varying durations yielded similar shape parameters, but with some variation in scale parameter, with the shortest duration tracks yielding the greatest scale parameters (Supplementary Fig. 15). It is possible that this observation reflects greater mobility and faster turnover for shorter filaments, as might be expected if filaments are restrained by crosslinks that are randomly and uniformly distributed along their lengths. However, it is also possible that this result may reflect experimental factors, for example the fact that brighter fluorophores were more likely to be successfully tracked over a long period of time than dim ones. To further probe this observation, we fit both stress fiber and cortical populations to the 1D jump model with reversals, including either all tracks or only long (> 14 s) tracks, and found that while there were changes in the best fit parameters, overall trends held (i.e. the cortical population was best fit by shorter time between jumps and higher reversal probability than tracks from stress fibers, Supplementary Table 2). Thus, despite some possible differences, the movement of both short- and long-duration tracks was qualitatively similar, and well-described by a jump model.

1. L Stirling Churchman, Zeynep Ökten, Ronald S Rock, John F Dawson, and James A Spudich. Single molecule high-resolution colocalization of cy3 and cy5 attached to macromolecules measures intramolecular distances through time. *Proceedings of the National Academy of Sciences*, 102(5):1419–1423, 2005.
2. Michael R Bubb, Ilan Spector, Bret B Beyer, and Katina M Fosen. Effects of jasplakinolide on the kinetics of actin polymerization: an explanation for certain in vivo observations. *Journal of Biological Chemistry*, 275(7):5163–5170, 2000.
3. Jonathan J Campbell and Martin M Knight. An improved confocal frap technique for the measurement of long-term actin dynamics in individual stress fibers. *Microscopy research and technique*, 70 (12):1034–1040, 2007.
4. Marco Fritzsche, Alexandre Lewalle, Tom Duke, Karsten Kruse, and Guillaume Charras. Analysis of turnover dynamics of the submembranous actin cortex. *Molecular biology of the cell*, 24(6): 757–767, 2013.
5. Matthew B Smith, Tai Kiuchi, Naoki Watanabe, and Dimitrios Vavylonis. Distributed actin turnover in the lamellipodium and frap kinetics. *Biophysical journal*, 104(1):247–257, 2013.
6. Mark Skamrahl, Huw Colin-York, Liliana Barbieri, and Marco Fritzsche. Simultaneous quantification of the interplay between molecular turnover and cell mechanics by afm–frap. *Small*, 15(40): 1902202, 2019.

Supplementary Table 1. Representative log10(likelihood-ratios) for several two-parameter model comparisons, from one experimental replicate. Abbreviations are as follows: Weibull: Wbl, folded Gaussian: Fgaus. Jump represents the 1D jump model, without reversals.

Population	Wbl vs. Fgaus		Jump vs. Rice		Wbl vs. Jump		Jump vs. Fgaus	
timescale	20 s	40 s	20 s	40 s	20 s	40 s	20 s	40 s
SFs	76	1.9	377	53	14	0.9	62	1.0
cortical	47	6.5	343	51	17	4.0	30	2.5
adhesions	11	5.3	71	2.5	4.1	4.0	6.7	1.2
adhesions$\wedge$SFs	3.8	1.9	45	11	2.1	0.9	1.7	0.9

Supplementary Table 2. Best-fit fit parameters for all tracks vs. only long tracks, fit to a 1D jump model with reversals. Data fit is from both stress fiber and cortical populations, from one experimental replicate.

	All tracks		Tracks > 14 s	
Fit parameter	Stress fibers	Cortical	Stress fibers	Cortical
average time between jumps (s)	6.9	3.6	8.9	5.6
average jump distance (nm)	54.5	61.5	49.4	61.3
reversal probability	0.26	0.37	0.03	0.19

Supplementary Table 3. Statistical comparison of the fit parameters of F-actin populations fit to the position jump model with reversals. P-values are from a Tukey comparison following 1-way ANOVA.

		ln(time between jumps)		jump distance		ln(reversal probability)	
Compared populations		est. diff. (s)	P-value	est. diff. (nm)	P-value	est. diff.	P-value
stress fibers	cortical	1.17	0.0092	19.7	0.0718	0.029	0.9999
stress fibers	adhesions	0.92	0.0454	20.5	0.0587	1.444	0.0104
stress fibers	stress fibers ∩ adhesions	0.42	0.5654	18.8	0.0905	1.654	0.0035
cortical	adhesions	-0.25	0.8535	0.79	0.9995	1.414	0.0121
cortical	stress fibers ∩ adhesions	-0.76	0.1186	-0.93	0.9993	1.625	0.0041
adhesions	stress fibers ∩ adhesions	-0.50	0.4095	-1.72	0.9954	0.211	0.9499

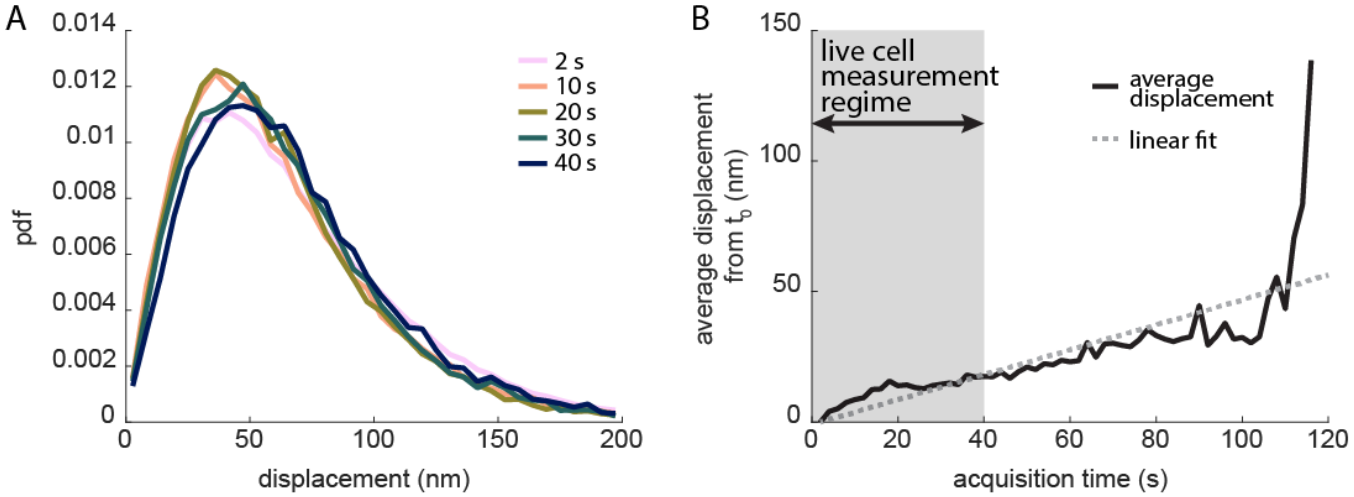

Supplementary Fig. 1. a. Displacement distributions in fixed cells measured over the 2, 10, 20, 30, and 40 s timescales. b. The net displacement vs. time, averaged across all long tracks (>=20 s) in n=9 fixed cells, and a best linear fit. The shaded region represents the timescale regime in which our live cell measurements are made. The displacement is normalized by subtracting the distance after 1 frame, which reflects primarily localization error. The steady increase in net distance is consistent with residual drift. Source data are provided in the Source Data file.

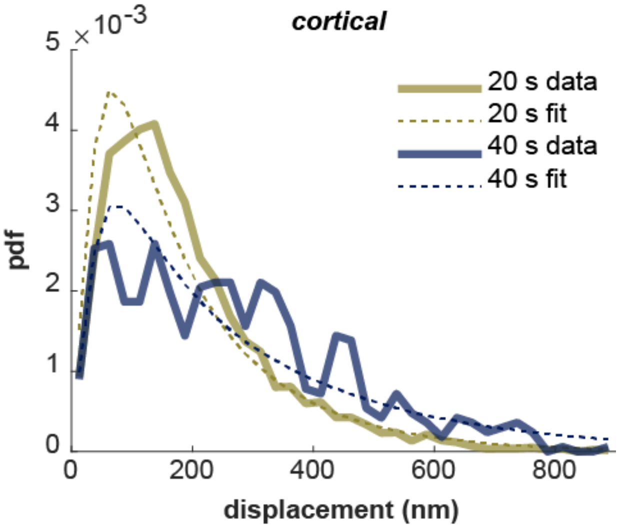

Supplementary Fig. 2. Exponential fits to the F-actin displacement distributions from the cortical population in HFFs, shown at 20 and 40 s timescales. n = 8 cells. Source data are provided in the Source Data file.

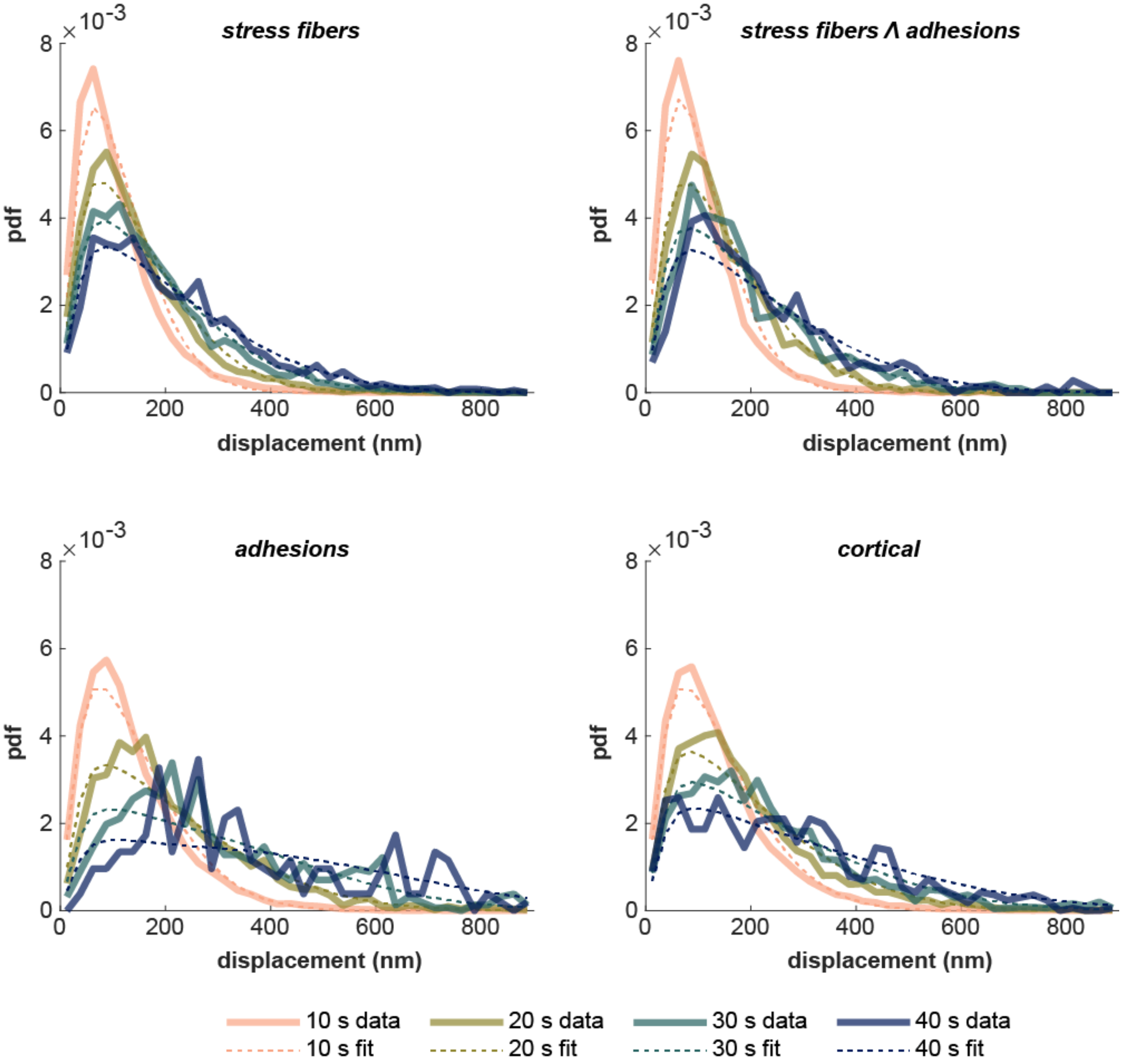

Supplementary Fig. 3. Folded Gaussian fits to F-actin displacement distributions from all four populations in HFFs, over varying timescales (10, 20, 30, and 40 s). The folded Gaussian tends to either underestimate the tails (most noticeable at the 10 s timescale) or offset the peak in order to fit the tails (e.g. the adhesion and cortical fits at longer timescales). n = 8 cells. Source data are provided in the Source Data file.

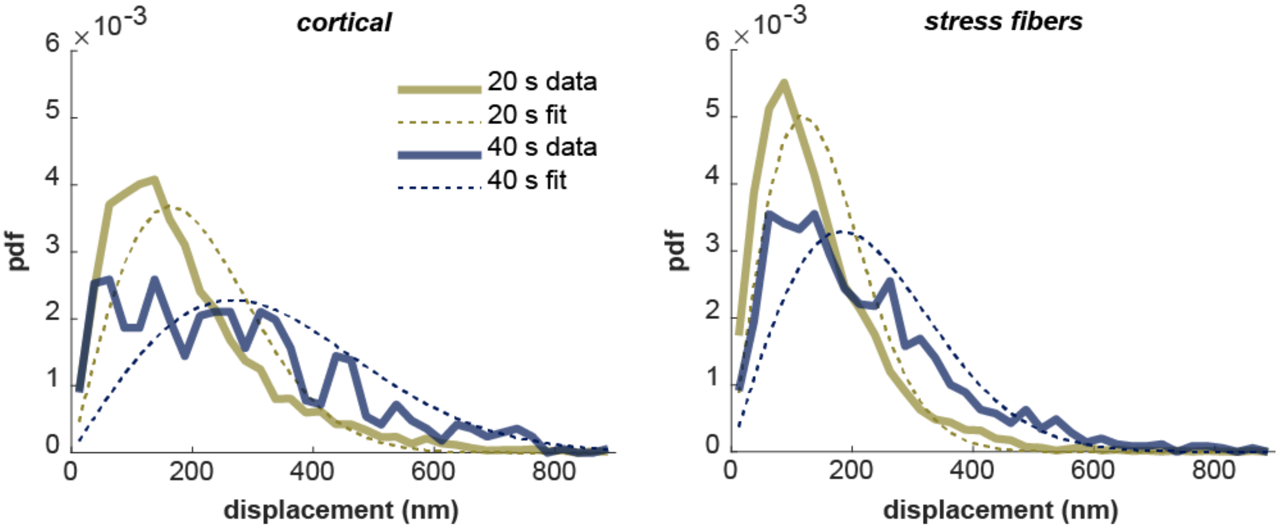

Supplementary Fig. 4. Rayleigh fits to F-actin displacement distributions from both stress fiber and cortical populations in HFFs at the 20 s and 40 s timescales. n = 8 cells. Source data are provided in the Source Data file.

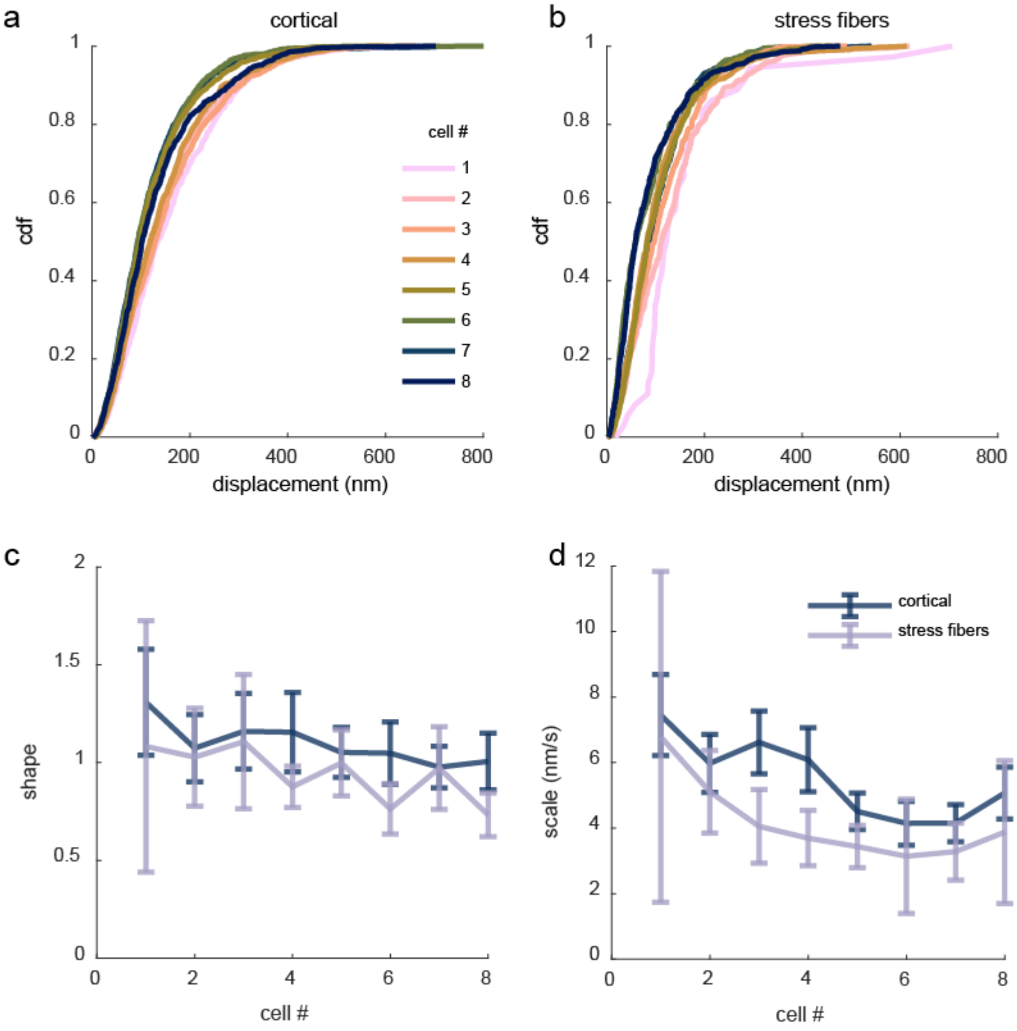

Supplementary Fig. 5. a,b) Cumulative distribution functions of raw (uncorrected) displacement distributions for cortical (a) and stress fiber (b) populations in HFFs for eight separate cells. All cdfs represent displacements over a 20 s time interval. c,d) Best fit parameters for a Weibull distribution fit to the displacement distributions in a and b. Center points mark the best fit parameters for data from each cell, and error bars show the 95% confidence interval for each parameter, determined by normal approximation (see Methods, Model Fitting). n = 8 cells. Source data are provided in the Source Data file.

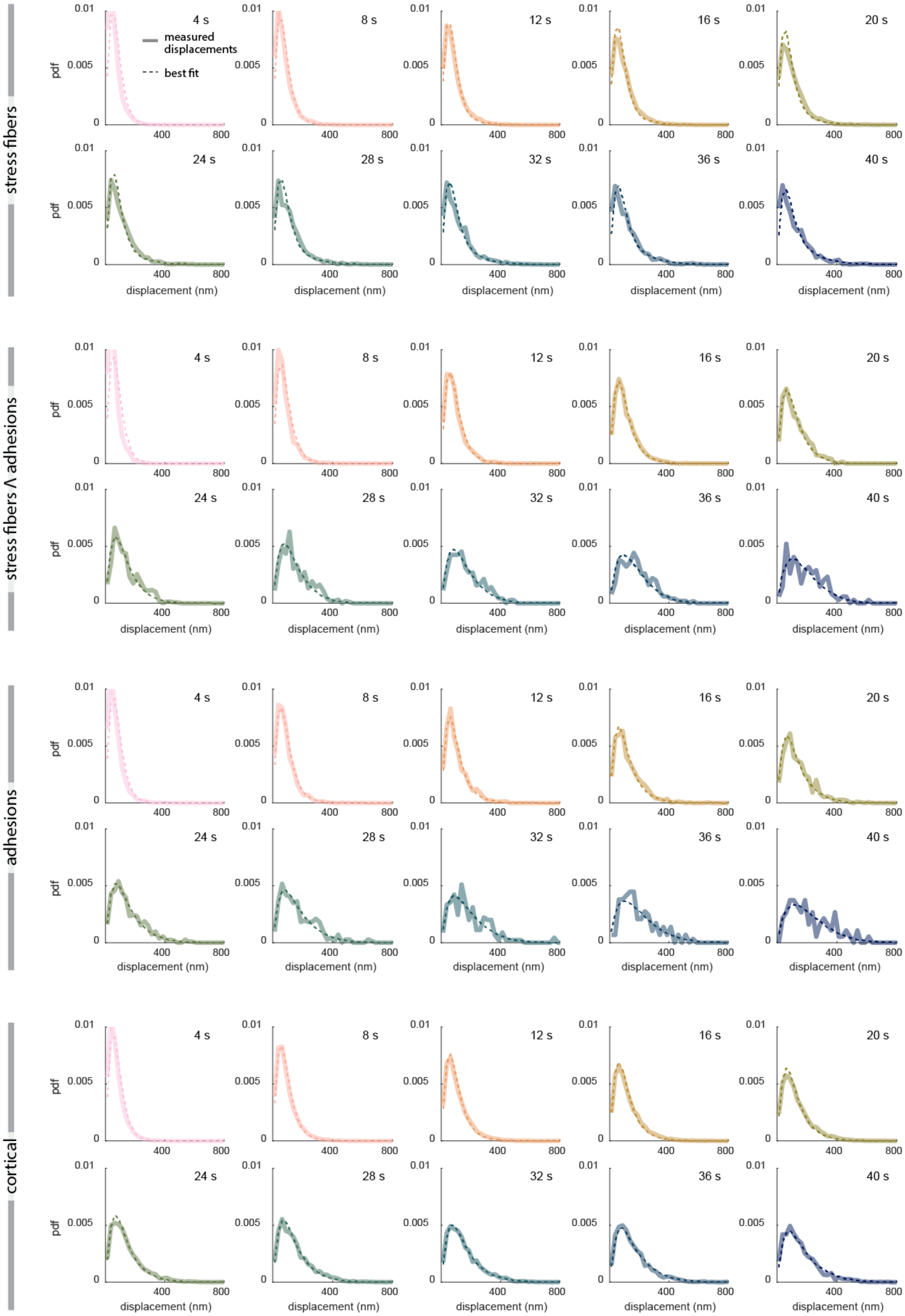

Supplementary Fig. 6. F-actin displacement distributions and the corresponding fits of the 1D position jump process with reversals for the four actin populations in HFFs, representative of 1 experiment with n=9 cells. All timescales are fit simultaneously by one set of fit parameters. Source data are provided in the Source Data file.

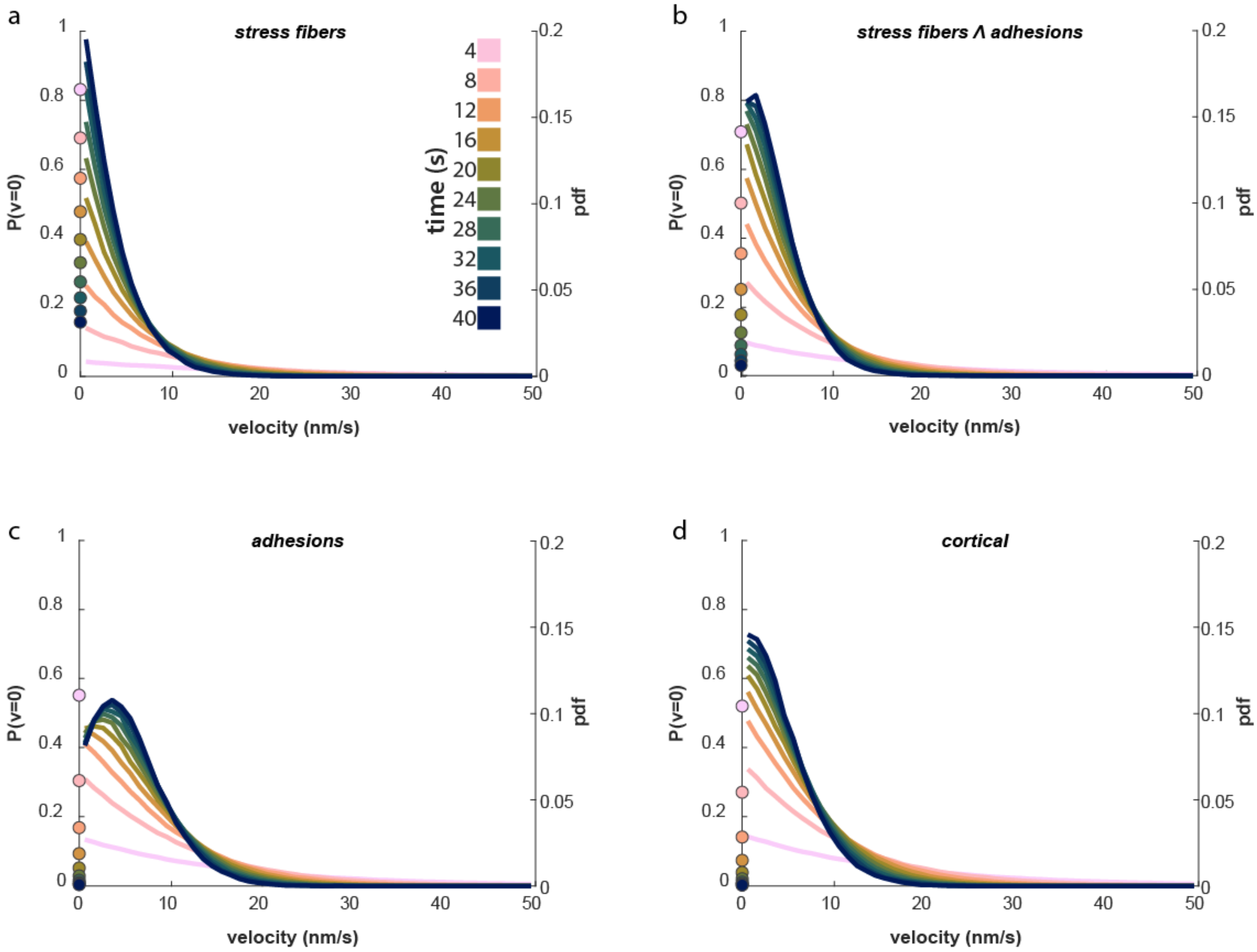

Supplementary Fig. 7. Inferred velocity distributions from the 1-D jump model with reversals for all four F-actin populations in HFFs: stress fibers (a), stress fibers and adhesions (b), adhesions (c), and cortical (d). The distributions for each cellular population are generated from the average best fit parameters (Fig 4g) across all HFF datasets (n = 32 total cells from 4 experimental replicates). In each case, the discrete probability of zero velocity is shown on the left y-axis, while the right y-axis gives the pdf values for the continuous pdf at velocities greater than zero. Source data are provided in the Source Data file.

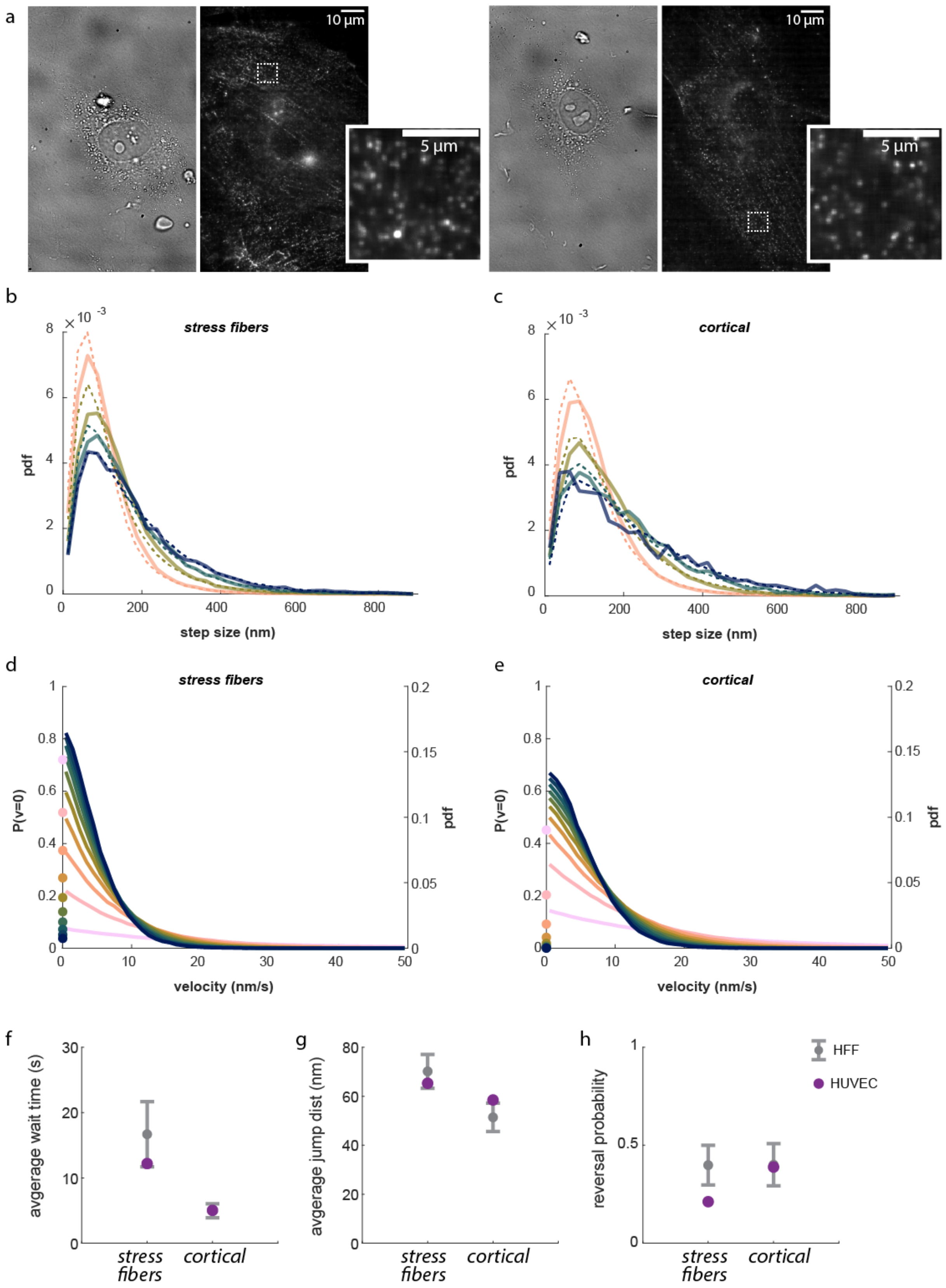

Supplementary Fig. 8. a) Representative images of two HUVECs, each shown in brightfield (left) and in the SiR-actin channel (right) after denoising with noise2void. Panels of zoomed in regions show the labelling density at the beginning of the acquisition. Images are representative of one experiment, n=8 cells. b, c) Measured probability density functions for stress fibers (b) and cortical actin (c) in HUVECs for varying timescales, and the corresponding best fit for each to the 1D position jump with reversals. d, e) The inferred velocity probability distributions for the fits to the distributions in (b) and (c). f, g, h) The best fit parameters from each fit for HUVECs (shown in b and c), as compared to those for HFFs. n = 8 cells. Source data are provided in the Source Data file.

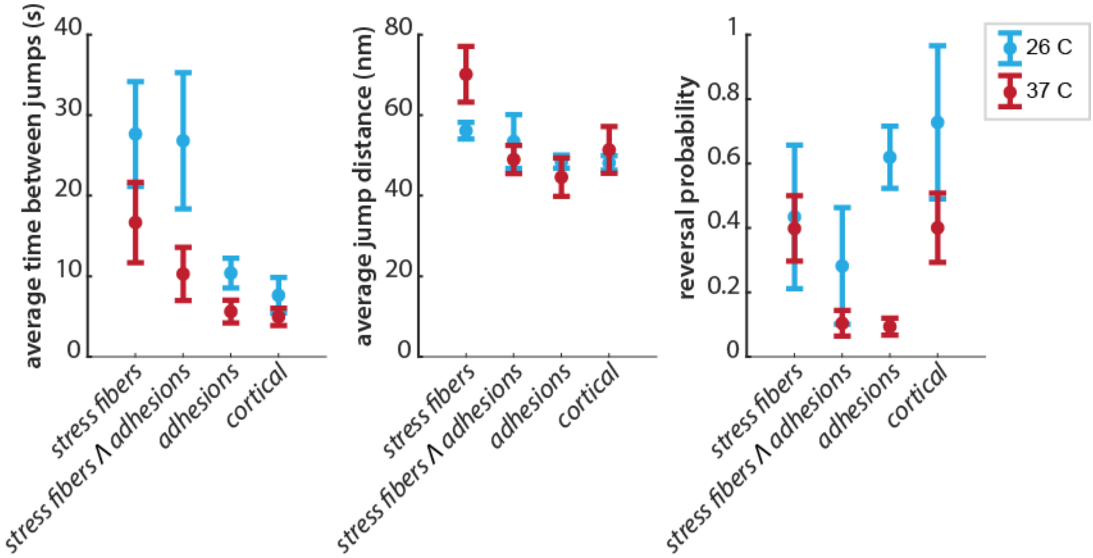

Supplementary Fig. 9. Temperature dependence of best fit parameters of the 1D position jump with reversals in HFFs. Measurements were made at both 37°C and 26°C. Points represent the average best fit parameters across 3-4 experiments (26°C, 3 experiments with n = 29 total cells; 37°C, 4 experiments with n = 32 total cells). Error bars represent the standard error on the mean from independent experiments. Source data are provided in the Source Data file.

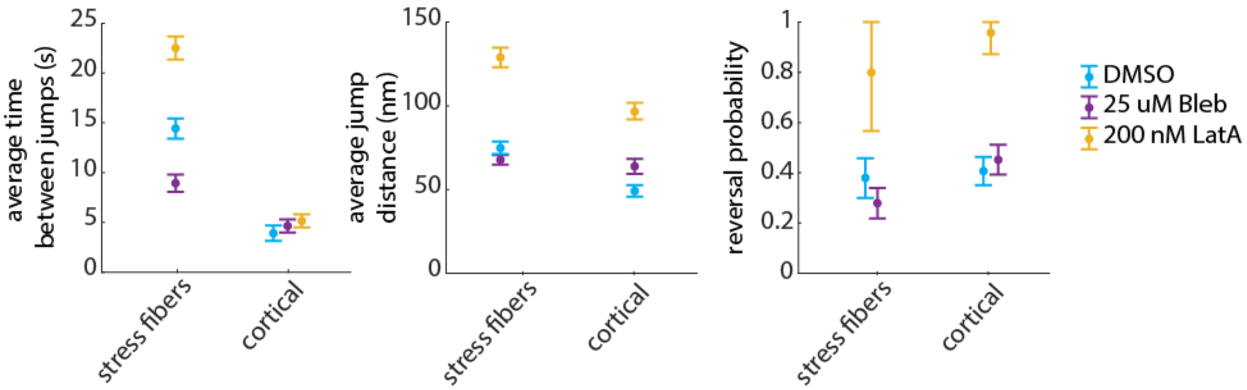

Supplementary Fig. 10. Best fit parameters of the 1D position jump with reversals to actin tracks in HFFs treated with 0.1% DMSO (n = 9 cells), 25 μM Blebistatin (n = 11 cells), or 200 nM Latrunculin A (n = 11 cells). Center points mark the best fit parameters for each population, and errorbars represent the 95% confidence intervals on each fit parameter, calculated by the profile likelihood method described in Methods: Model Fitting. Source data are provided in the Source Data file.

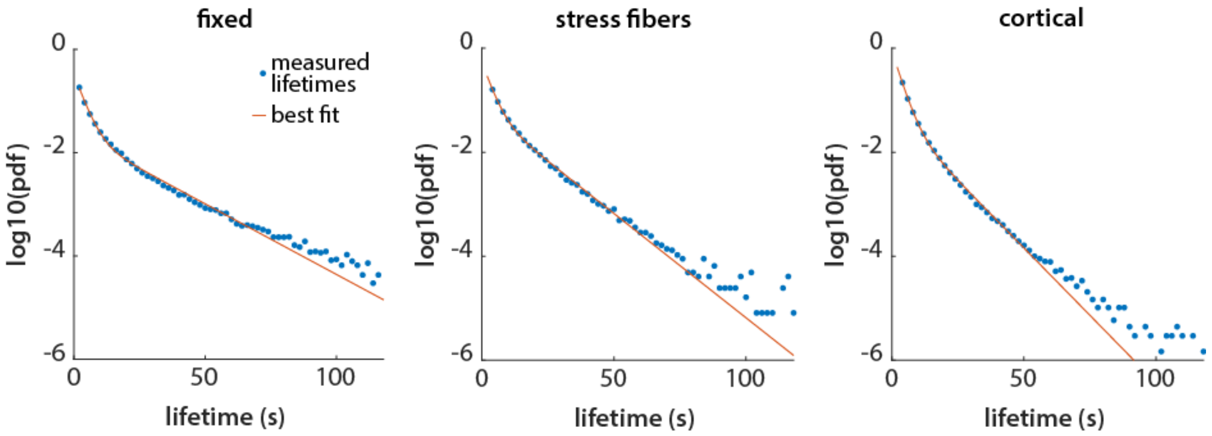

Supplementary Fig. 11. Measured lifetimes of SiR-actin speckles in fixed cells (left, n = 10 cells), and live cell stress fiber (middle) and cortical (right) populations (n = 9 cells), as well as biexponential fits as described in Supplementary Note 4. Source data are provided in the Source Data file.

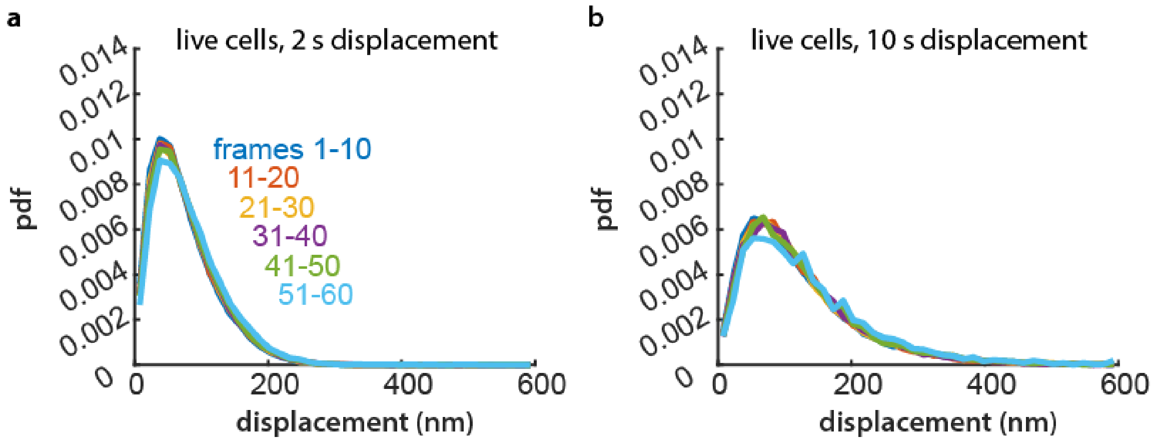

Supplementary Fig. 12. Measured actin speckle displacements over varying portions of a movie. Displacements over a 2 s time interval (a) and a 10 s time interval (b) in live cells (all subcellular populations), measured during varying portions of a movie (frames 1-10, 11-20, . . . , 51-60). n = 9 cells. Source data are provided in the Source Data file.

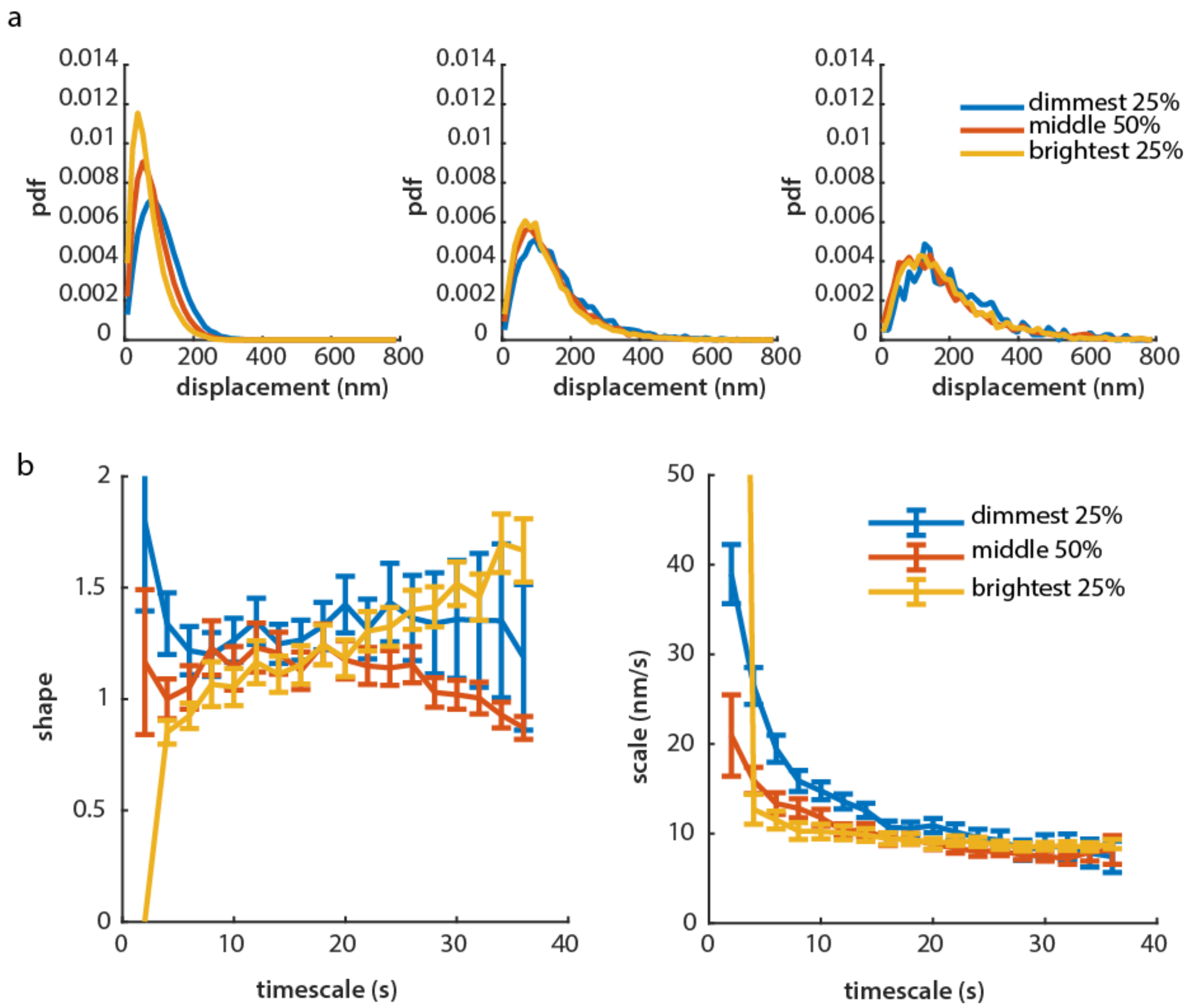

Supplementary Fig. 13. Displacements of tracks with varying punctum brightness and Weibull fits to the same. a) Measured displacements at three timescales (2 s, left; 10 s, center; and 20 s, right) for the dimmest 25%, the brightest 25%, and the remaining 50% of tracked puncta. b) Best-fit Weibull parameters to the same populations, across timescales from 2 s to 36 s. Center points mark the best fit parameters for data from each cell, and error bars show the 95% confidence interval for each parameter, determined by normal approximation (see Methods, Model Fitting). These data represent the cortical population from one experimental replicate, n = 9 cells. Source data are provided in the Source Data file.

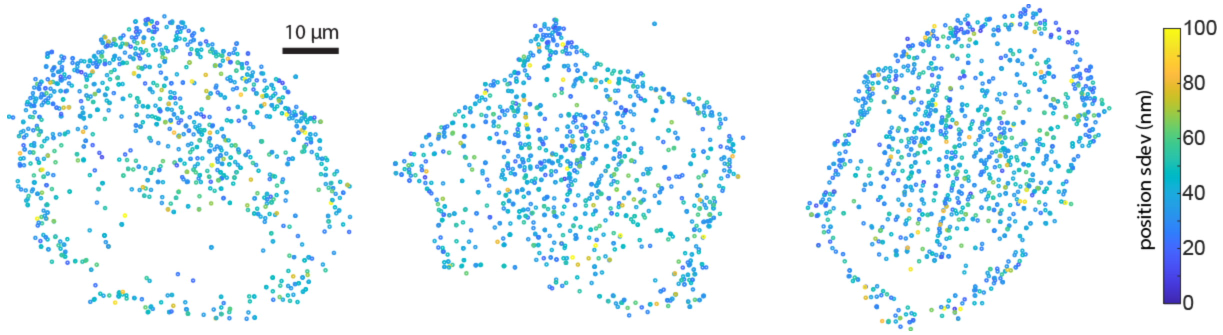

Supplementary Fig. 14. Three representative fixed cells, where each track with at least 5 subpixel localizations is shown. Tracks are represented by a spot centered at their mean position, and are color-coded by the standard deviation of their positions (averaged over x and y). Source data are provided in the Source Data file.

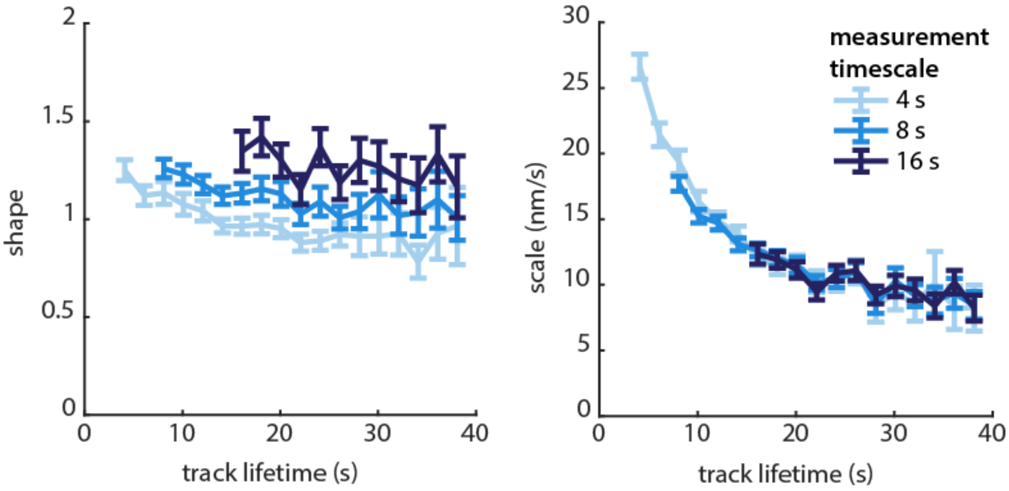

Supplementary Fig. 15. Best-fit Weibull parameters for tracks of varying durations, measured at three time intervals: 4 s, 8 s, and 16 s. Center points mark the best fit parameters for data from each cell, and error bars show the 95% confidence interval for each parameter, determined by normal approximation (see Methods, Model Fitting). These data are drawn from the cortical population from one experimental replicate, n = 9 cells. Source data are provided in the Source Data file.

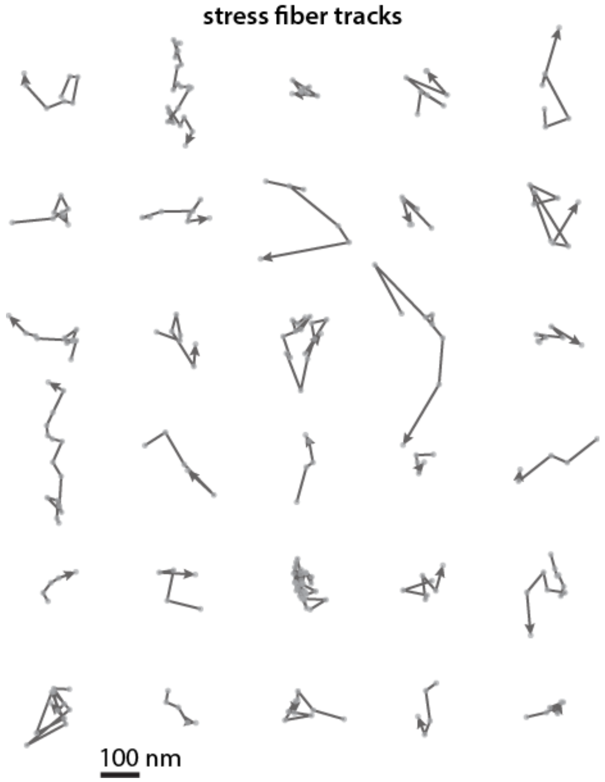

Supplementary Fig. 16. Examples of several long tracks (>= 5 localizations) from stress fibers in live cells. Source data are provided in the Source Data file.
