# Supplementary material for: Extraction of accurate cytoskeletal actin velocity distributions from noisy measurements
Source: Nat Commun. 2022 Aug 13;13:4749. doi: 10.1038/s41467-022-31583-y (PMC9376101; doi:10.1038/s41467-022-31583-y)
Supplement: Supplementary file 3 — Description of Additional Supplementary Files [file 41467_2022_31583_MOESM3_ESM.pdf]

Description of Additional Supplementary Files

File Name: Supplementary Movie 1
Description: **An HFF labeled with a low dose of SiR-actin.** Left: the whole cell is imaged every 2 s for 120 s total. The scale bar is 10 µm. Right: the two boxed regions of the cell on the left, magnified 6x. The scale bar is 1 µm.

File Name: Supplementary Movie 2
Description: **Speckle tracking by QFSM and subpixel localization.** An HFF labeled with SiR-actin, imaged every 2 s for 120 s total, overlaid with tracked speckles from QFSM (circles). Localizations which pass subpixel localization are shown in green; those that fail are shown in magenta (see Methods, Actin tracking analysis for details). Video has been denoised using noise2void.

File Name: Supplementary Movie 3
Description: **A HUVEC labeled with a low dose of SiR-actin.** The labeled cell is imaged every 2 s for 3 min 16 s total. The scale bar is 10 µm.
