# Supplementary material for: Extraction of accurate cytoskeletal actin velocity distributions from noisy measurements
Source: Nat Commun. 2022 Aug 13;13:4749. doi: 10.1038/s41467-022-31583-y (PMC9376101; doi:10.1038/s41467-022-31583-y)
Supplement: Supplementary file 7 — Reporting Summary [file 41467_2022_31583_MOESM7_ESM.pdf]

nature portfolio

Corresponding author(s): Alexander R Dunn

Last updated by author(s): June 3 2022

Reporting Summary

Nature Portfolio wishes to improve the reproducibility of the work that we publish. This form provides structure for consistency and transparency in reporting. For further information on Nature Portfolio policies, see our Editorial Policies and the Editorial Policy Checklist.

Statistics

For all statistical analyses, confirm that the following items are present in the figure legend, table legend, main text, or Methods section.

n/a	Confirmed	
☐	☒	The exact sample size (n) for each experimental group/condition, given as a discrete number and unit of measurement
☐	☒	A statement on whether measurements were taken from distinct samples or whether the same sample was measured repeatedly
☐	☒	The statistical test(s) used AND whether they are one- or two-sided<br>*Only common tests should be described solely by name; describe more complex techniques in the Methods section.*
☒	☐	A description of all covariates tested
☐	☒	A description of any assumptions or corrections, such as tests of normality and adjustment for multiple comparisons
☐	☒	A full description of the statistical parameters including central tendency (e.g. means) or other basic estimates (e.g. regression coefficient) AND variation (e.g. standard deviation) or associated estimates of uncertainty (e.g. confidence intervals)
☐	☒	For null hypothesis testing, the test statistic (e.g. F, t, r) with confidence intervals, effect sizes, degrees of freedom and P value noted<br>*Give P values as exact values whenever suitable.*
☒	☐	For Bayesian analysis, information on the choice of priors and Markov chain Monte Carlo settings
☒	☐	For hierarchical and complex designs, identification of the appropriate level for tests and full reporting of outcomes
☒	☐	Estimates of effect sizes (e.g. Cohen's d, Pearson's r), indicating how they were calculated

Our web collection on statistics for biologists contains articles on many of the points above.

Software and code

Policy information about availability of computer code

Data collection	Micro-Manager (v1) was used for collection of microscopy data in this study.
Data analysis	Quantitative Fluorescent Speckle Microscopy (QFSM, v1.0.1) was used to produce tracks from microscopy videos. Fiji Is Just ImageJ (v1.53c) was used to generate masks for subcellular population grouping. Noise2Void (v0.8.4, TensorFlow v1.15) was used to denoise microscopy images. NanoJ (Nov 2018 version) was used to generate drift tables for microscopy videos. Matlab (v2020b, including Curve Fitting, Global Optimization, Image Processing, Optimization, Parallel Computing, and Statistics and Machine Learning toolboxes) was used for subpixel localization, generation of distributions, and all model fitting.

For manuscripts utilizing custom algorithms or software that are central to the research but not yet described in published literature, software must be made available to editors and reviewers. We strongly encourage code deposition in a community repository (e.g. GitHub). See the Nature Portfolio guidelines for submitting code & software for further information.

Data

Policy information about availability of data

All manuscripts must include a data availability statement. This statement should provide the following information, where applicable:

- Accession codes, unique identifiers, or web links for publicly available datasets
- A description of any restrictions on data availability
- For clinical datasets or third party data, please ensure that the statement adheres to our policy

The microscopy data and corresponding tracked puncta generated in this study have been deposited in the Zenodo database under doi https://doi.org/10.5281/zenodo.6609641

Field-specific reporting

Please select the one below that is the best fit for your research. If you are not sure, read the appropriate sections before making your selection.

☒ Life sciences ☐ Behavioural & social sciences ☐ Ecological, evolutionary & environmental sciences

For a reference copy of the document with all sections, see nature.com/documents/nr-reporting-summary-flat.pdf

Life sciences study design

All studies must disclose on these points even when the disclosure is negative.

Sample size	Sample sizes were not chosen a priori, but for each experimental condition/replicate, 7-12 cells were analyzed. We completed a number of measures of robustness of our measurements, including comparing the results from multiple cells within a sample, and find there to be relatively little cell-to-cell variation. Each cell yields on the order of tens of thousands of tracked speckles.
Data exclusions	Data exclusion was largely performed at the imaging step. Cells with low signal of either GFP-paxillin or SiR-actin, such that adhesions or actin structures could not be distinguished, were excluded from the data collection.
Replication	For the experimental data that are included in the main text, 4 experimental replicates were included. While F-actin velocities varied between replicates, trends and main conclusions (e.g. cortical actin shows greater displacements than stress fiber actin) were consistent across replicates.<br>Experimental replication occasionally failed at the cell staining step due to failure of the SiR-actin staining (often correlated with SiR-actin reagent age after reconstitution >1 month)--in these cases, images were not collected and analyzed.
Randomization	Cells in experimental groups were random subsets of the same cell stock. Cells were seeded into several wells of a coverwell chamber, or on multiple slides. Slides/wells were chosen randomly for each experimental group.
Blinding	Investigators were not blinded to the experimental groups.<br>During data collection, the differences between groups were obvious (e.g. fixed and live cells easily distinguished from one another by lack of movement), and certain perturbations (i.e. temperature control and actin perturbations) were necessarily performed at the microscope during or immediately preceding data collection, making blinding difficult.<br>During analysis, experimental and control conditions were evaluated using the same mathematical formalisms and code.

Reporting for specific materials, systems and methods

We require information from authors about some types of materials, experimental systems and methods used in many studies. Here, indicate whether each material, system or method listed is relevant to your study. If you are not sure if a list item applies to your research, read the appropriate section before selecting a response.

Materials & experimental systems

n/a	Involved in the study
☒	☐ Antibodies
☐	☒ Eukaryotic cell lines
☒	☐ Palaeontology and archaeology
☒	☐ Animals and other organisms
☒	☐ Human research participants
☒	☐ Clinical data
☒	☐ Dual use research of concern

Methods

n/a	Involved in the study
☒	☐ ChIP-seq
☒	☐ Flow cytometry
☒	☐ MRI-based neuroimaging

Eukaryotic cell lines

Policy information about cell lines

Cell line source(s)	HFFs were from ATCC (CCD-1070Sk); HUVECs were from Lonza (C2519A)
Authentication	Cell lines were not authenticated.
Mycoplasma contamination	Cell lines were not tested for mycoplasma.
Commonly misidentified lines (See ICLAC register)	No commonly misidentified lines were used in the study.
